# Integrated analysis of tumor-associated macrophages and M2 macrophages in CRC: unraveling molecular heterogeneity and developing a novel risk signature

**DOI:** 10.1186/s12920-024-01881-z

**Published:** 2024-05-27

**Authors:** Lujing Shi, Hongtun Mao, Jie Ma

**Affiliations:** Department of Gastroenterology Surgery, Shengli Oilfield Central Hospital, Dgongying, Shandong P. R. China

**Keywords:** Tumor-associated macrophages, M2 macrophages, Colorectal cancer, scRNA-seq, Risk signature

## Abstract

**Background:**

Emerging investigations have increasingly highlighted the critical role of tumor-associated macrophages (TAMs) and M2 macrophages in cancer development, progression, and metastasis, marking them as potential targets in various cancer types. The main objective of this research is to discover new biomarkers associated with TAM-M2 macrophages in colorectal cancer (CRC) and to dissect the molecular heterogeneity of CRC by combining single-cell RNA sequencing and bulk RNA-seq data.

**Methods:**

By utilizing weighted gene co-expression network analysis (WGCNA), we acquired TAM-M2-associated genes by intersecting TAM marker genes obtained from scRNA-seq data with module genes of M2 macrophages derived from bulk RNA-seq data. We employed least absolute shrinkage and selection operator (LASSO) Cox analysis to select predictive biomarkers from these TAM-M2-related genes. Quantitative polymerase chain reaction (qPCR) was employed to validate the mRNA expression levels of the genes identified in the screening. This led to the development of the TAM-M2-related signature (TAMM2RS). We also conducted functional and immune landscape analyses of different risk groups.

**Results:**

The combination of scRNA-seq and bulk RNA-seq analyses yielded 377 TAM-M2-related genes. DAPK1, NAGK, and TRAF1 emerged as key prognostic genes in CRC, which were identified through LASSO Cox analysis. Utilizing these genes, we constructed and validated the TAMM2RS, demonstrating its effectiveness in predicting survival in CRC patients.

**Conclusion:**

Our research offers a thorough investigation into the molecular mechanisms associated with TAM-M2 macrophages in CRC and unveils potential therapeutic targets, offering new insights for treatment strategies in colorectal cancer.

**Supplementary Information:**

The online version contains supplementary material available at 10.1186/s12920-024-01881-z.

## Background

Colorectal cancer (CRC) remains a major global health concern, with an estimated 2 million new diagnoses and approximately 900,000 deaths in 2020 [1]. Moreover, CRC’s diverse clinical and molecular profiles exhibit marked differences in tumor progression and therapeutic responsiveness [2]. However, the pathogenic pathways driving CRC, though intricate, remain only partially elucidated. Hence, this situation highlights an exigent need for comprehensive investigation endeavors and the development of novel signatures to refine our predictive capabilities for CRC patient outcomes.

Myeloid cells emerge as a dominant immune subset within TME, involved in a spectrum of roles from immunosuppressive to immunostimulatory activities [3]. Notably, tumor-associated macrophages (TAMs) delineate a dynamic subpopulation, displaying a plasticity that enables phenotypic transitions contingent on TME cues [3]. The traditional dichotomy of macrophages into pro-inflammatory M1 and pro-tumoral M2 subsets has undergone a foundational shift [4–6]. Advances in single-cell RNA sequencing (scRNA-Seq) technologies have illuminated a more delicate macrophage spectrum, revealing overlapping transcriptional gene expression profiles between M1 and M2 entities [7–9]. While TAMs are conspicuously absent under normal physiological conditions, their presence in various tumors has prompted reconsideration of their classification. Intriguingly, while TAMs exhibit characteristics reminiscent of both M1 and M2 polarization, their operational functionalities mirror M2 macrophages [10]. Their pivotal roles in modulating TME immune landscapes, predominantly through immune suppression and facilitation of tumor immune evasion, accentuate their significance [11]. Furthermore, the paramountcy of TAMs in the TME crystallizes their potential as therapeutic targets, underscoring the imperative for in-depth insights into TAM-M2-mediated CRC pathogenesis and the consequent development of associated prognostic signatures.

**Fig. 1:**
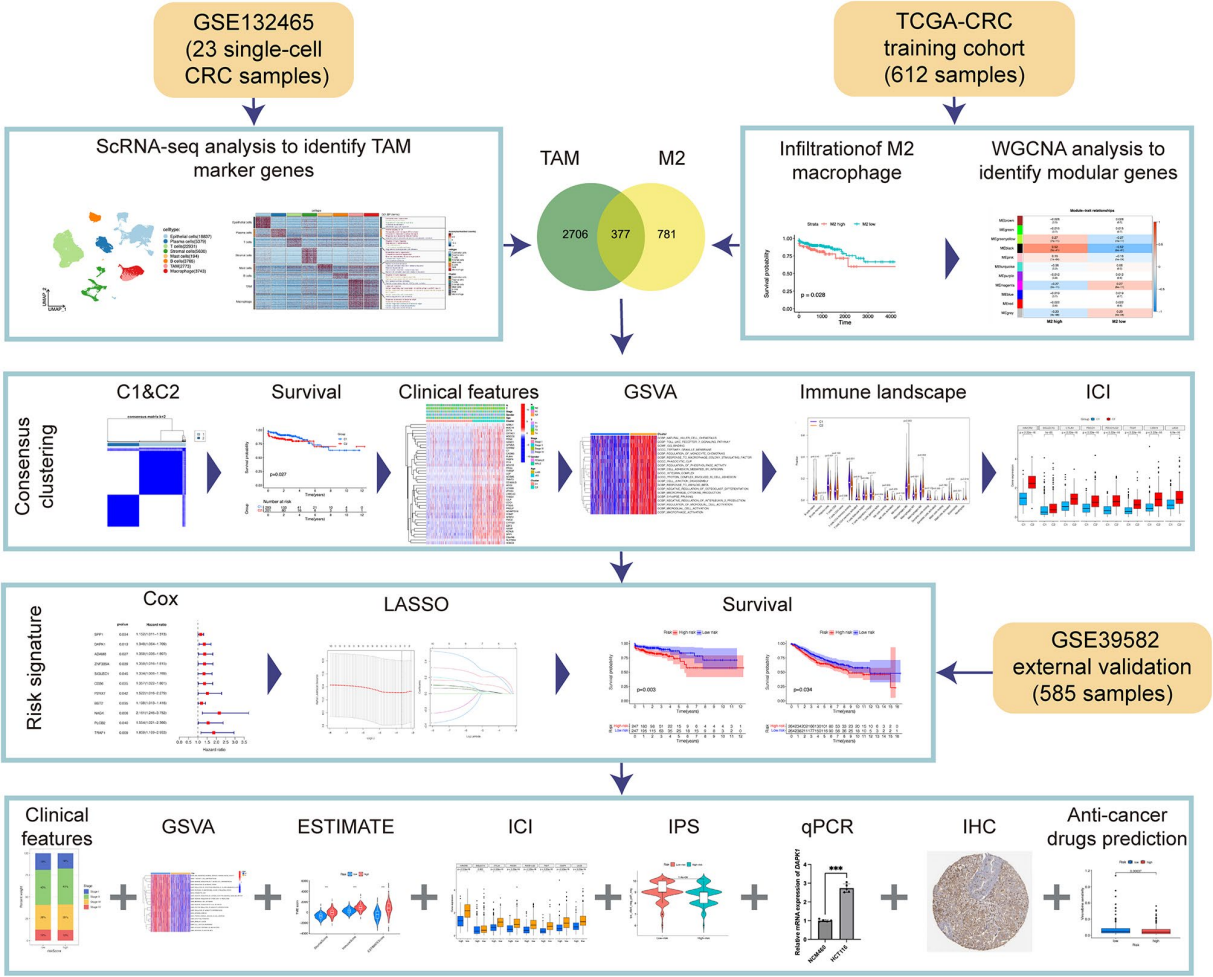
Workflow of the study

Here, we collaboratively employ the scRNA-seq and bulk RNA-seq datasets to delineate the molecular heterogeneity of CRC based on marker genes of M2-TAMs. Then, we introduced a TAM-M2-related signature (TAMM2RS) for CRC. The integrative approach is illustrated in Fig. 1.

## Methods

### Data curation

The single-cell data (GSE132465) were obtained from the Gene Expression Omnibus (GEO) database. The bulk RNA-seq data (including clinical data) were retrieved from The Cancer Genome Atlas (TCGA-CRC, *n* = 612) and GEO (GSE39582, *n* = 585) repositories.

### Analyses of macrophage infiltration

Utilizing the CIBERSORT method, we evaluated the infiltration of M2 macrophages across TCGA-CRC samples. The optimal threshold for distinguishing high and low infiltration of M2 macrophages was established by the survminer package. Employing the survival package, we analyzed survival differences between sub-groups specified by high and low M1/M2 macrophage infiltration. By stratifying CRC specimens into categories based on their M2 macrophage infiltration (high or low), we conducted the Weighted Gene Co-expression Network Analysis (WGCNA) [12]. The objective of this analysis was to identify genes closely associated with M2 macrophage infiltration. We performed a clustering of the samples to evaluate their collective significance within the dataset, while excluding any outliers. Guided by the point where the scale-free topology fit index showed a substantial value, the selection of the soft-thresholding power β was determined at the minimum power. We set the minimum threshold for genes per module at 60.

### ScRNA-seq data processing

We conducted the processing of the scRNA-seq dataset using Seurat package (version 4.3.0) [13]. The quality control criteria: preserving cells that have more than 300 identified genes and genes expressed in over three cells; excluding cells with > 20% mitochondrial gene expression. After cell filtration, normalization of the high-quality cellular data was performed, identifying highly variable genes pivotal for subsequent steps. Principal component analysis (PCA) was then applied to these genes to determine key principal components (PCs). For the visualization of cell clusters, we employed the Uniform Manifold Approximation and Projection (UMAP) method. Following this, the FindAllMarkers function was instrumental in identifying marker genes specific to each cluster. Each cell type was subsequently annotated, drawing references from the CellMarker 2.0 database (http://bio-bigdata.hrbmu.edu.cn/CellMarker/). The FeatureHeatmap function was employed to illustrate the distinctiveness of each cell type and its corresponding biological processes.

### Intersection of TAM and M2 macrophage associated genes

We executed correlation assessments between modules and specific traits to identify modules that significantly correlate with high M2 macrophage infiltration. Subsequently, the genes within these identified modules were cross-referenced with TAM marker genes, which were derived from scRNA-seq data analyses.

### Consensus clustering

Initially, we conducted a Cox regression analysis to identify TAM-M2 genes with potential prognostic value, which were then used for consensus clustering analysis utilizing the ConsensusClusterPlus package [14]. The optimal cluster count was ascertained by examining the cumulative distribution function (CDF) and its delta area. The prognostic relevance of our clustering was corroborated by constructing K-M survival curves, using the survminer package. Furthermore, we employed the limma package to identify differentially expressed genes (DEGs) across clusters, focusing on those with an absolute log fold change (|logFC|) > 1 and an adjusted P-value < 0.05 [15].

### Functional analyses

The DEGs identified were subsequently incorporated into a Gene Ontology (GO) functional analysis. Following this, we presented a comprehensive heatmap to depict the expression patterns and clinicopathological features of screened genes across the various clusters. Moreover, to elucidate the distinct biological profiles, we employed the GSVA (Gene Set Variation Analysis) package [16]. This approach facilitated a precise assessment of the unique biological attributes inherent to each identified cluster.

### Immune landscape

To illustrate the abundance of 23 different types of immune cells across distinct clusters and explore the TME, we performed CIBERSORT and then employed the ggplot2 package for visualization [17]. Furthermore, to uncover potential targets for CRC immunotherapy, we examined the variability in expression of human leukocyte antigen (HLA) and immune checkpoint inhibitor (ICI) genes across the clusters.

### Construction of TAMM2RS

We employed Cox regression analyses using the glmnet package on genes that were common to both TAM from scRNA-seq data and M2 macrophages from bulk RNA-seq data, with the aim of identifying prognostic genes. Following this, we employed LASSO Cox regression analysis to ascertain the coefficients of each gene that held predictive value. Based on these coefficients, risk scores were computed:

$$\varvec{R}\varvec{i}\varvec{s}\varvec{k} \varvec{s}\varvec{c}\varvec{o}\varvec{r}\varvec{e}={\sum }_{\varvec{i}=1}^{\varvec{n}}\left(\mathbf{c}\mathbf{o}\mathbf{e}\mathbf{f}\mathbf{i}*\mathbf{e}\mathbf{x}\mathbf{p}\mathbf{i}\right)$$

Based on median risk score, CRC samples were categorized into either a low-risk group (LRG) or a high-risk group (HRG). This categorization enabled the generation of K-M curves, which were used to underscore the survival differences between the risk groups. To further corroborate the robustness of this risk signature, the GSE39582 dataset was included to form an external validation cohort.

Moreover, the variation in clinicopathological features was delineated using the ggplot2 package. Furthermore, the survival disparities between the HRG and LRG were stratified and analyzed in the context of age, gender, and clinical stage.

### GSEA

Utilizing the clusterProfiler package, we conducted gene set enrichment analysis (GSEA) to investigate the pathways that are predominantly enriched in HRG [18, 19]. These pathways were ordered based on their normalized enrichment scores, and the most significant pathways were selected for detailed visualization.

### Immune infiltration analysis

We employed the Estimation of Stromal and Immune cells in Malignant Tumor tissues using Expression data (ESTIMATE) analysis to assess immune cell infiltration. This approach quantified stromal and immune scores, as well as the overall ESTIMATE scores, within the tumor microenvironment. Moreover, we utilized several algorithms to evaluate the correlation between risk score and macrophage infiltration.

### Prediction of immunotherapy response

We sourced immunophenotype data from The Cancer Immunome Atlas to predict how CRC samples would respond to immunotherapy treatments. This data was utilized to compute the immunophenoscore (IPS) for each sample.

### Cell culture

Human CRC cell line HCT116 (CL-0096) was obtained from Procell Life Science & Technology (China), while the NCM460 (iCell-h373) cell line, representing normal colonic epithelial cells, was acquired from Cellverse (China). HCT116 cells were propagated in DMEM (C11995500BT, Gibco, USA), and NCM460 cells were cultivated in RPMI 1640 medium (10-040-CV, Corning, USA). Both media were fortified with 10% fetal bovine serum (FBS; C0235, Gibco, USA) and a combination of 100 U/ml penicillin and 100 µg/ml streptomycin (C0222, Beyotime, China). Cell cultures were incubated at 37 °C in a humidified incubator with a 5% CO2 atmosphere.

### Quantitative polymerase chain reaction (qPCR)

qPCR was employed to quantify mRNA expression levels utilizing the EZBioscience™ PCR array (EZBioscience, Roseville, CA, USA). Gene expression quantification was conducted applying the comparative 2^(-ΔΔCt) method, normalizing to GAPDH as the endogenous control. This analysis was independently replicated on three separate occasions. Primer sequences utilized for amplification are detailed in Table 1.

**Table 1:** Primers of qPCR

Primer	Forward (5’ to 3’)	Reverse (5’ to 3’)
NAGK	CACTATTTCCAGGTGCCAG	GAAGATATAGCGGGAAAGGG
DAPK1	CATCAAGAACCGAGAAGGAG	CAATGTGTCCGTCCTTGTC
TRAF1	AAAGAGAACCCATCTGTCG	ATGAAGGTGACCTTGTTCC
GAPDH	TCAAGATCATCAGCAATGCC	CGATACCAAAGTTGTCATGGA

### Immunohistochemistry (IHC) analysis

We verified the protein expression profiles of both normal and CRC samples through the Human Protein Atlas (HPA) database.

### Anti-cancer drugs prediction

Utilizing the oncoPredict package, our study assessed the anticancer effectiveness by gauging drug sensitivity in CRC patients [20].

### Statistical analyses

R software (version 4.3.1) was employed to conduct the statistical analyses. Survival time distributions were estimated via the K-M method. The Wilcoxon test was utilized to compare two cohorts, while the Kruskal-Wallis test was carried out to examine differences among multiple groups. Statistical significance was indicated by establishing a P-value threshold below 0.05.

## Results

### Acquisition of genes related to M2 macrophages

**Fig. 2:**
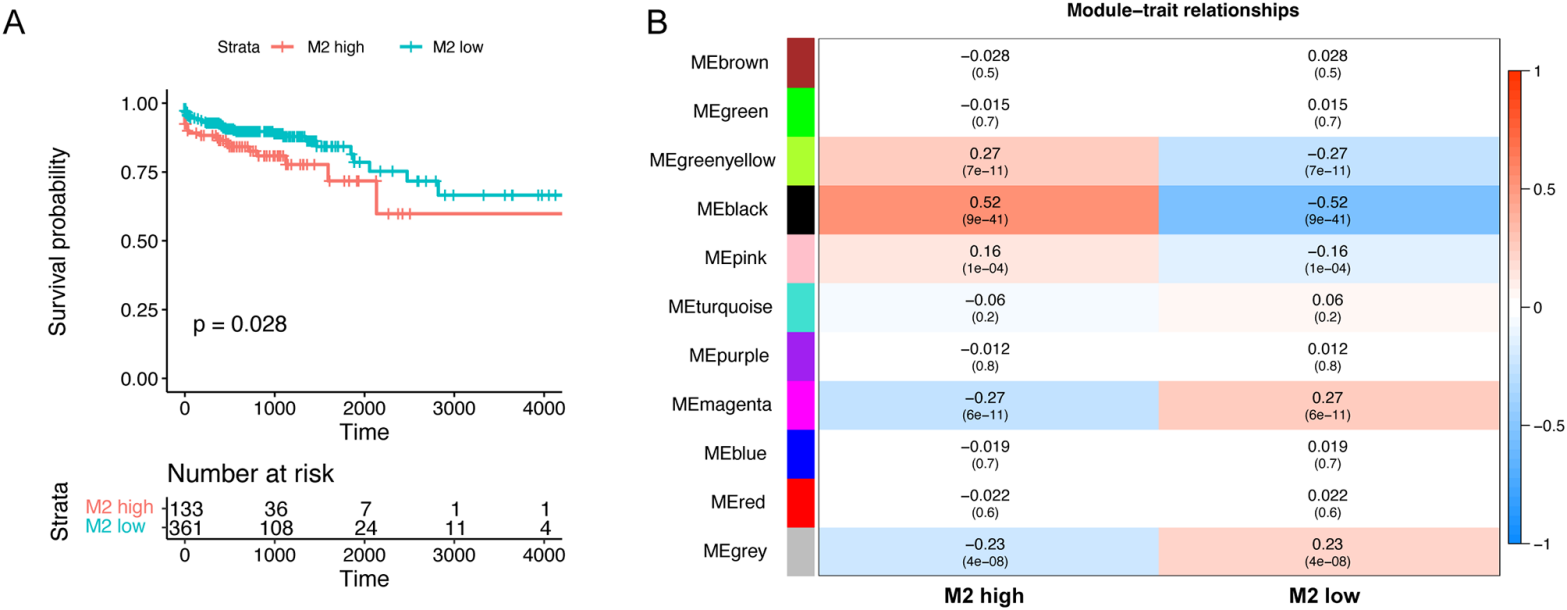
Identification of M2 macrophage-related genes. (**A**) The group with high infiltration of M2 macrophages exhibited a worse prognosis. (**B**) WGCNA was utilized to identify M2 macrophage-related modular genes. WGCNA, weighted gene co-expression network analysis

Our investigation sought to elucidate the prognostic impact of macrophages in CRC. Patients from the TCGA-CRC dataset were stratified into groups with high or low M2 macrophage infiltration via CIBERSORT method. Survival analysis using the K-M approach revealed that patients characterized by a high infiltration of M2 macrophages exhibited reduced survival rates (Fig. 2A). This finding implicates M2 macrophages as significant prognostic factors in CRC. Subsequent WGCNA identified gene modules associated with M2 macrophage levels in CRC, with 11 modules emerging from the analysis (Fig. 2B, Figure S1). From these, the black module, containing 1,158 genes, was chosen for further analysis (Table S1).

### Identifying TAM marker genes

**Fig. 3:**
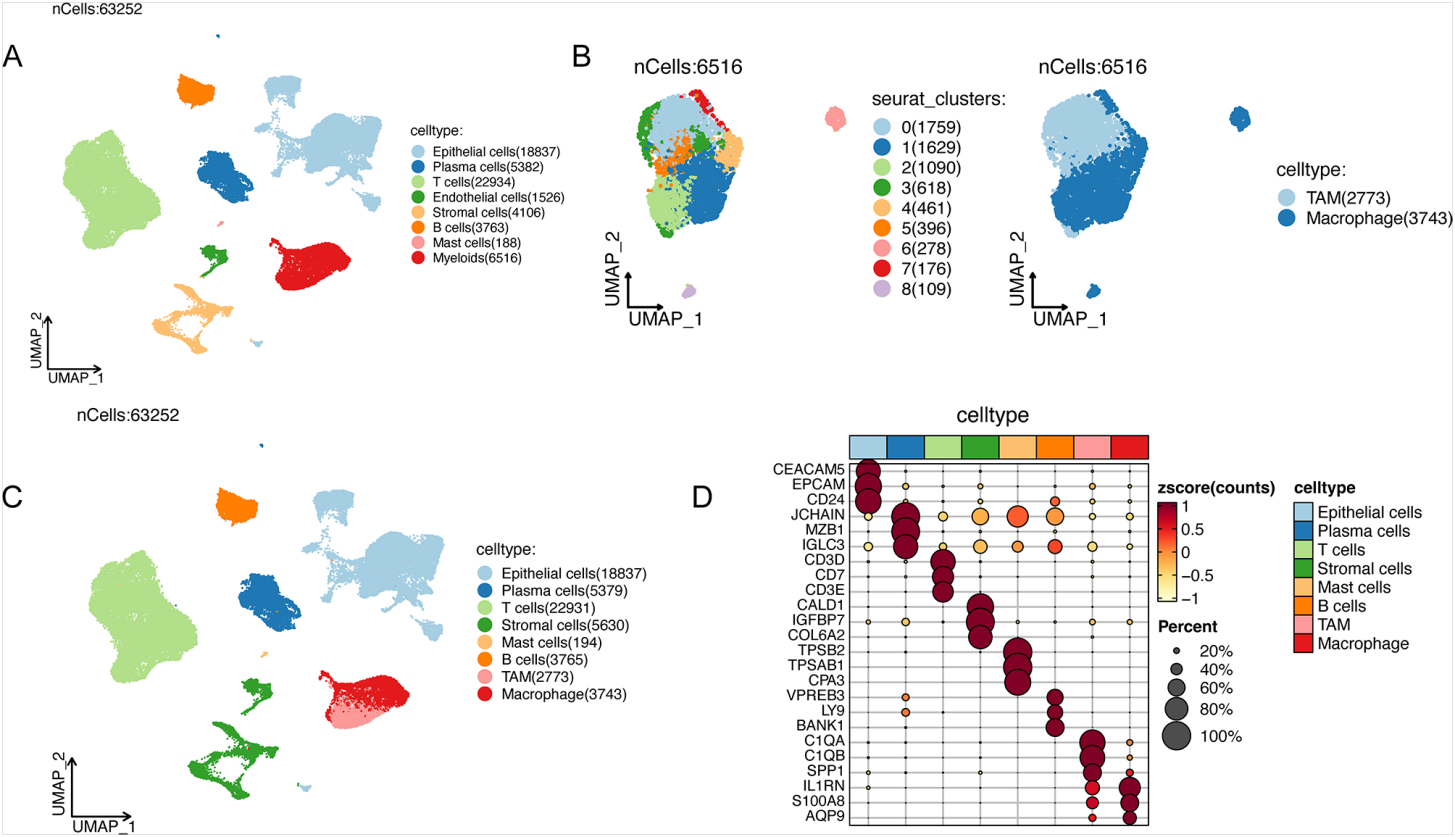
ScRNA-seq processing. (**A**) Cell annotation. (**B**) Myeloids was re-clustered and annotated. (**C**) Identification of TAM marker genes. (**D**) Distribution and expression of marker genes. TAM, tumor-associated macrophage

We analyzed the scRNA-seq data to identify TAM marker genes in CRC, with the objective of mapping the composition of the TME. After rigorous quality control and data normalization, a cohort of 63,252 cells from 23 CRC specimens was curated for subsequent analysis. Utilizing the FindNeighbors and FindClusters functions, we classified the cells into 39 distinct clusters (Figure S2). These clusters were subsequently annotated with cell type identities using the CellMarker 2.0 database, identifying diverse cellular populations including Epithelial cells, Plasma cells, T cells, Endothelial cells, Stromal cells, B cells, Mast cells, and Myeloids (Fig. 3A). Further refinement of the myeloid population allowed us to re-cluster these cells into 9 distinct clusters, which were categorized as TAMs and macrophages based on extant literature (Fig. 3B). 3,083 marker genes characteristic of TAMs were thus elucidated (Fig. 3C, Table S2). Additionally, the distribution and expression of marker genes for each cell type were illustrated in Fig. 3D.

**Fig. 4:**
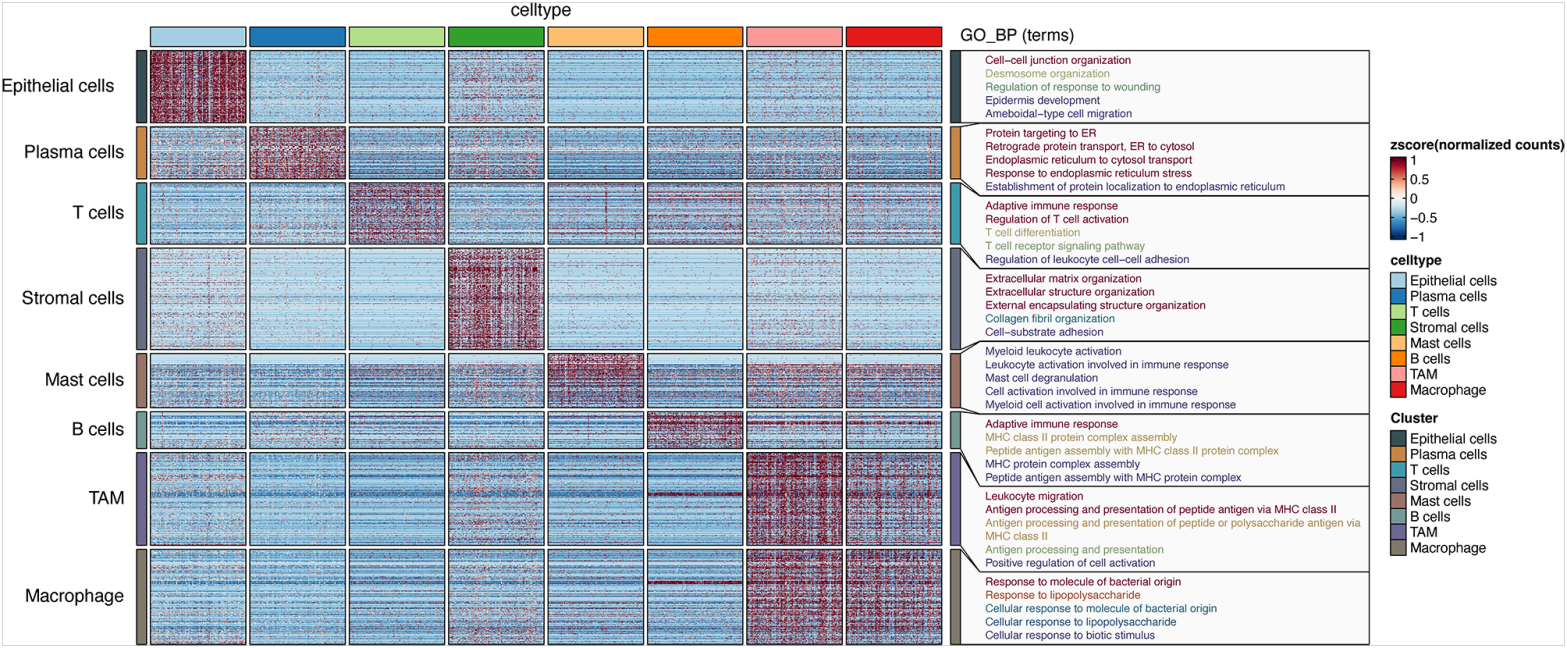
Heatmap displaying marker genes and biological processes for each cell type

Furthermore, we employed a heatmap for the validation of our annotation outcomes, which delineates the array of enriched biological processes alongside each cellular annotation, as illustrated in the rightmost column (Fig. 4).

### Determination of TAM-M2-mediated clusters

**Fig. 5:**
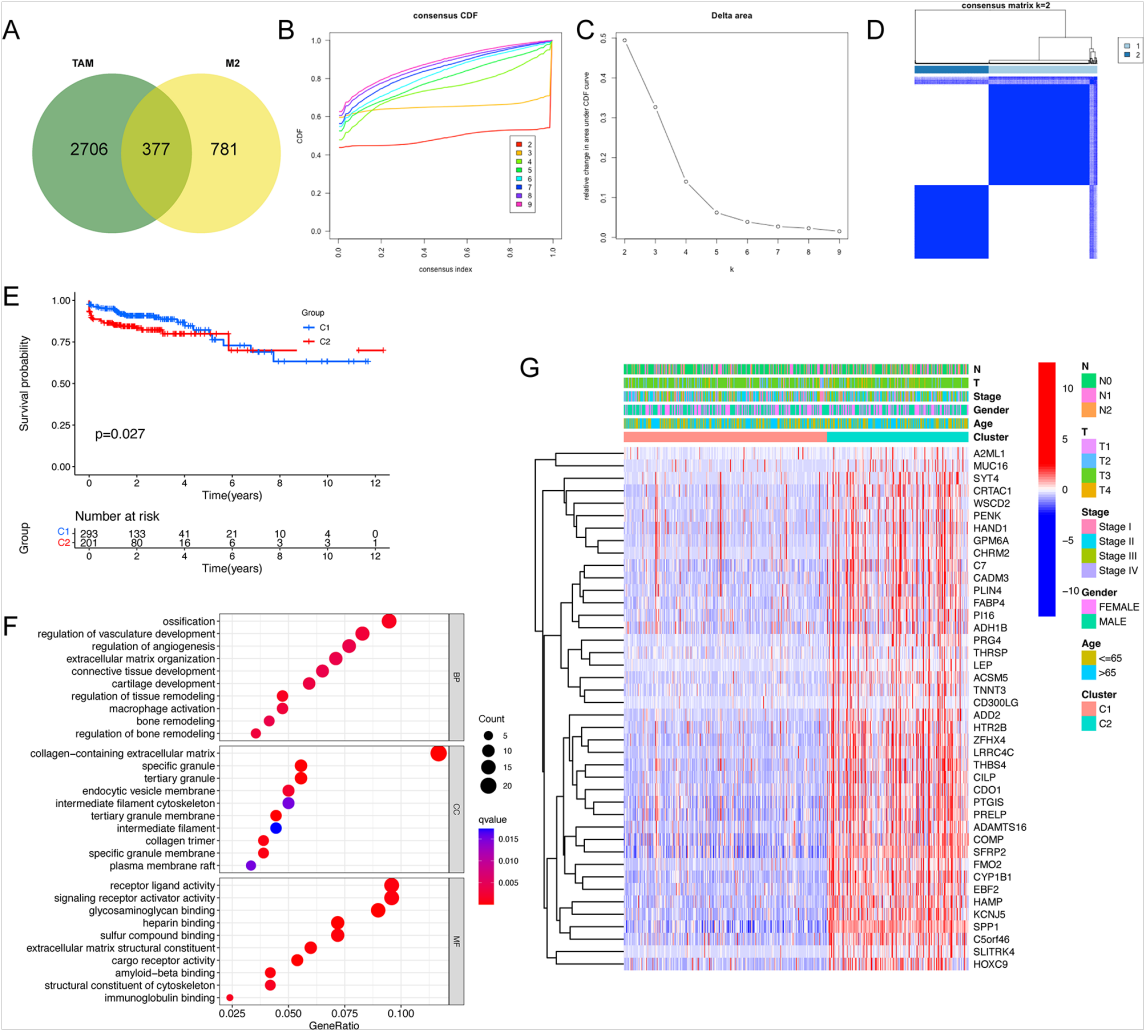
Clustering. (**A**) Intersection of TAM marker genes and M2 macrophage-related modular genes. (**B-D**) Unsupervised consensus clustering. (**E**) K-M survival analysis between C1 and C2. (**F**) GO analysis for DEGs between C1 and C2. (**G**) Distribution of clinical features and expression of prognostic DEGs. GO, Gene Ontology; DEGs, differentially expressed genes

Upon merging the gene sets pertaining to M2 macrophages with TAM marker genes, we retrieved a total of 377 genes associated with TAM-M2 macrophages (Fig. 5A, Table S3). To elucidate the TAM-M2-mediated heterogeneity within CRC, we applied consensus clustering to stratify the TCGA-CRC dataset into two principal clusters based on the expression profiles of TAM-M2-related genes (Fig. 5B–D). Notably, patients classified under cluster C2 were observed to have an adverse prognosis relative to those in cluster C1 (Fig. 5E).

Additionally, we incorporated the DEGs from clusters C1 and C2 into GO enrichment analysis. The findings revealed that these DEGs were predominantly enriched in biological processes related to ossification, regulation of vascular development, angiogenesis, extracellular matrix organization, and macrophage activation (Fig. 5F). Furthermore, we generated a heatmap to portray the distribution of significant genes identified by univariate Cox analysis and the differential clinicopathological features between clusters C1 and C2 (Fig. 5G).

### GSVA

**Fig. 6:**
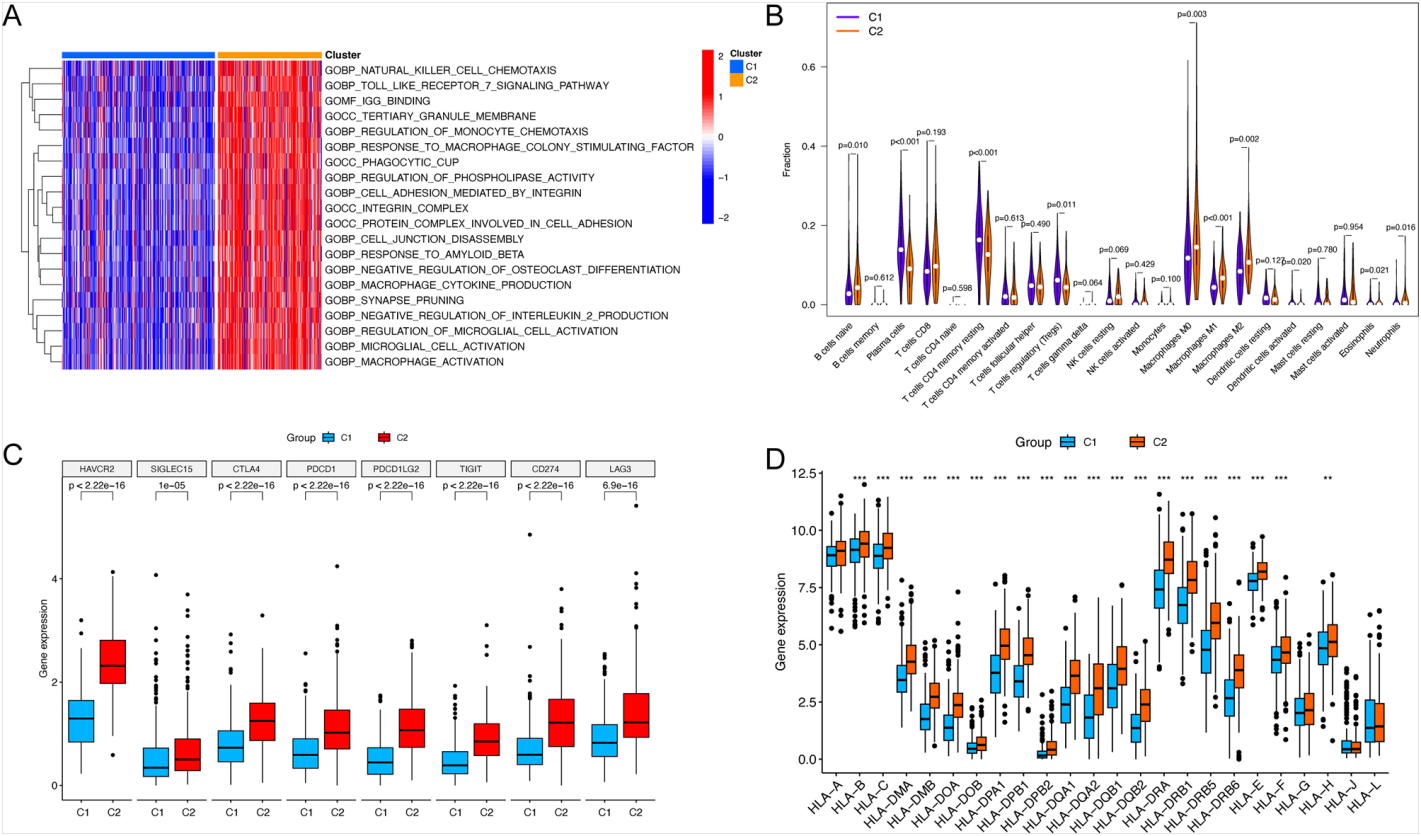
Functional and immune analyses between C1 and C2. (**A**) Heatmap illustrating GSVA differences between C1 and C2. (**B**) Immune infiltration analysis of 23 immune cells. (**C-D**) Difference in major ICI- and HLA-related genes between clusters. GSVA, gene set variation analysis; ICI, immune checkpoint inhibitor; HLA, human leukocyte antigen. *P < 0.05; **P < 0.01; ***P < 0.001

The findings from GSVA revealed a pronounced enrichment of C2 in several key biological processes, including natural killer cell chemotaxis, Toll-like receptor 7 signaling pathway, regulation of monocyte chemotaxis, macrophage cytokine production, macrophage activation and response to macrophage colony-stimulating factor (Fig. 6A).

### Immune analysis

The analysis using CIBERSORT demonstrated a notable infiltration of a majority of the 23 immune cell types in C2 (Fig. 6B). Following this, the expression patterns of ICI- and HLA-related genes were comparatively analyzed across the two clusters. The expression of genes related to ICI and HLA exhibited distinctive patterns across the two clusters. (Figs. 6C, D).

### Construction of TAMM2RS

**Fig. 7:**
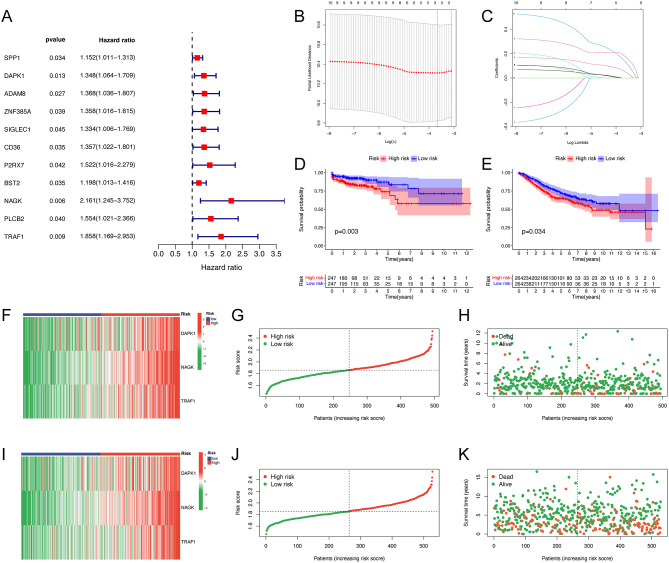
Construction of TAMM2RS. (**A**) Univariate Cox analysis for screened genes. (**B**-**C**) LASSO Cox regression analysis. K-M survival analyses for TCGA-CRC cohort (**D**) and GSE39582 cohort (**F**). Distribution of gene expressions, risk scores and survival statuses for TCGA-CRC cohort (**F-H**) and GSE39582 cohort (**I-K**). TAMM2RS, tumor-associated macrophages and M2 macrophages related signature; LASSO, least absolute shrinkage and selection operator; TCGA, The Cancer Genome Atlas; CRC, colorectal cancer

Utilizing the intersecting genes, we first applied univariate Cox regression analysis to screened genes of significant prognostic importance (Fig. 7A). Subsequently, these genes were incorporated into a LASSO Cox regression analysis. Through this process, we identified three key genes - DAPK1, NAGK, and TRAF1 - for the construction of the TAMM2RS (Fig. 7B, C, Table S4). The TCGA-CRC dataset was utilized as the training set, while the GSE39582 served as the validation set for external verification. K-M survival analysis across these cohorts indicated a less favorable outcome for the HRG (Fig. 7D, E). Additionally, Fig. 7F-K illustrated the variance in gene expression, risk scores, and survival statuses for both the training and external validation sets.

**Fig. 8:**
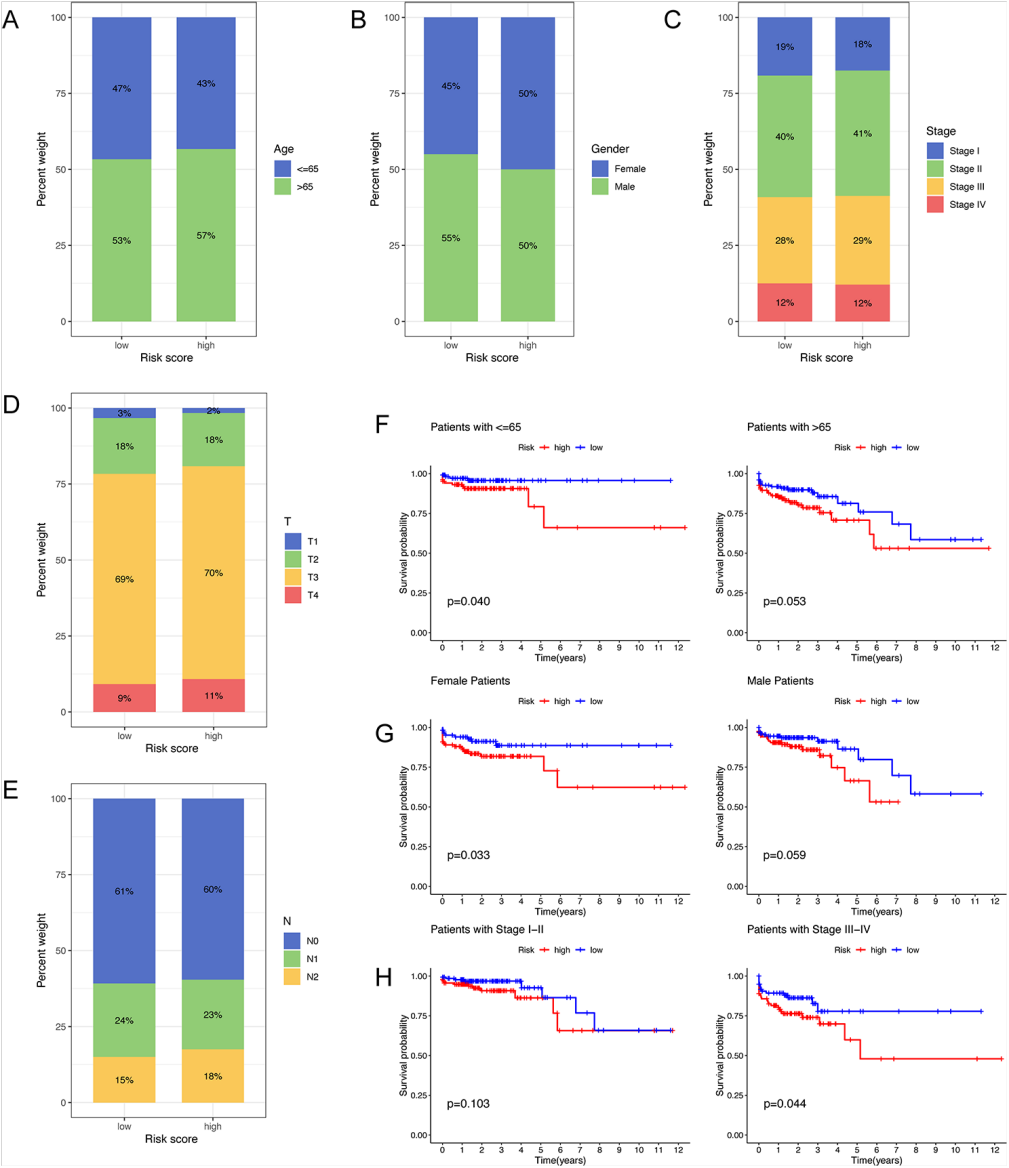
Distribution of clinical features and survival analyses. (**A-E**) Distribution of different clinical features between HRG and LRG. K-M survival analyses for stratified by age (**F**), gender (**G**) and stage (**H**). HRG, high-risk group; LRG, low-risk group

Additionally, we performed a sub-stratification analysis comparing the HRG and LRG. This analysis revealed a slight predominance of older age and advanced tumor stages within the HRG (Fig. 8A-E). K-M survival analysis further elucidated that patients in the HRG had a less favorable prognosis across both age groups (≤ 65 years and > 65 years) and in both genders (male and female), as shown in Fig. 8F and G. Notably, there was a significant prognostic difference in stages III-IV between HRG and LRG, whereas no such difference was apparent in stages I-II (Fig. 8H).

### Functional analyses between HRG and LRG

**Fig. 9:**
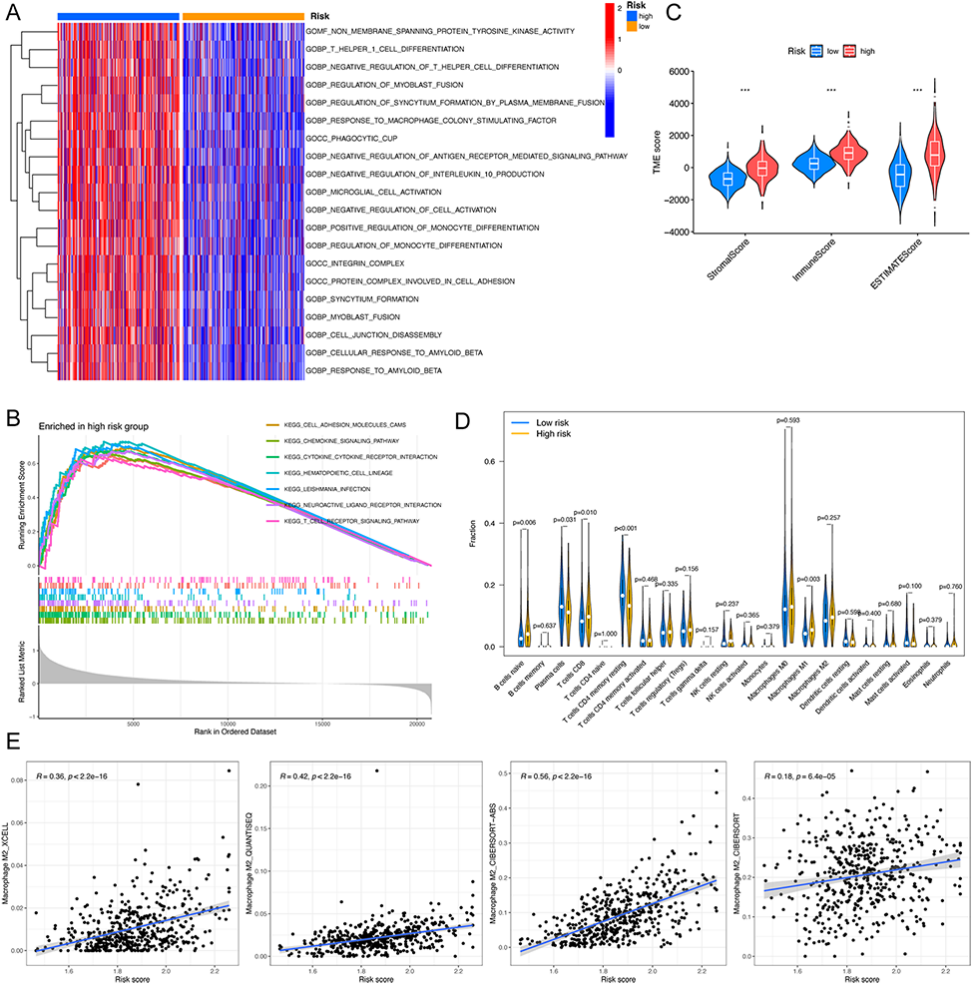
Functional and immune analyses between HRG and LRG. (**A**) Heatmap depicting GSVA differences between C1 and C2. (**B**) GSEA for HRG. (**C**) ESTIMATE analysis. (**D**) Immune infiltration analysis of 23 immune cells. (**E**) Correlation between risk score and M2 macrophage infiltration. HRG, high-risk group; LRG, low-risk group; GSVA, gene set variation analysis; GSEA, gene set enrichment analysis; ESTIMATE, Estimation of STromal and Immune cells in MAlignant Tumor tissues using Expression data. *P < 0.05; **P < 0.01; ***P < 0.001

GSVA disclosed that the HRG exhibited enrichment in several biological processes, notably T helper 1 cell differentiation, positive regulation of monocyte differentiation, response to macrophage colony-stimulating factor and regulation of monocyte differentiation (Fig. 9A). Furthermore, GSEA highlighted that the HRG predominantly showed enrichment in pathways related to cell adhesion molecules (CAMs), T cell receptor signaling pathway, cytokine-cytokine receptor interaction and chemokine signaling pathway (Fig. 9B).

### Immune investigations between HRG and LRG

The findings from ESTIMATE analyses revealed that, compared to the LRG, the HRG consistently showed higher scores in immune, stromal, and overall ESTIMATE scores, suggesting significant distinctions in the TME (Fig. 9C). Moreover, the HRG was characterized by a marginally higher immune infiltration relative to the LRG (Fig. 9D). To further investigate the relationship between risk score and M2 macrophage infiltration, we applied four distinct algorithms – XCELL, QUANTISEQ, CIBERSORT-ABS, and CIBERSORT. These analyses confirmed a positive correlation between the infiltration of M2 macrophages and the risk score (Fig. 9E).

**Fig. 10:**
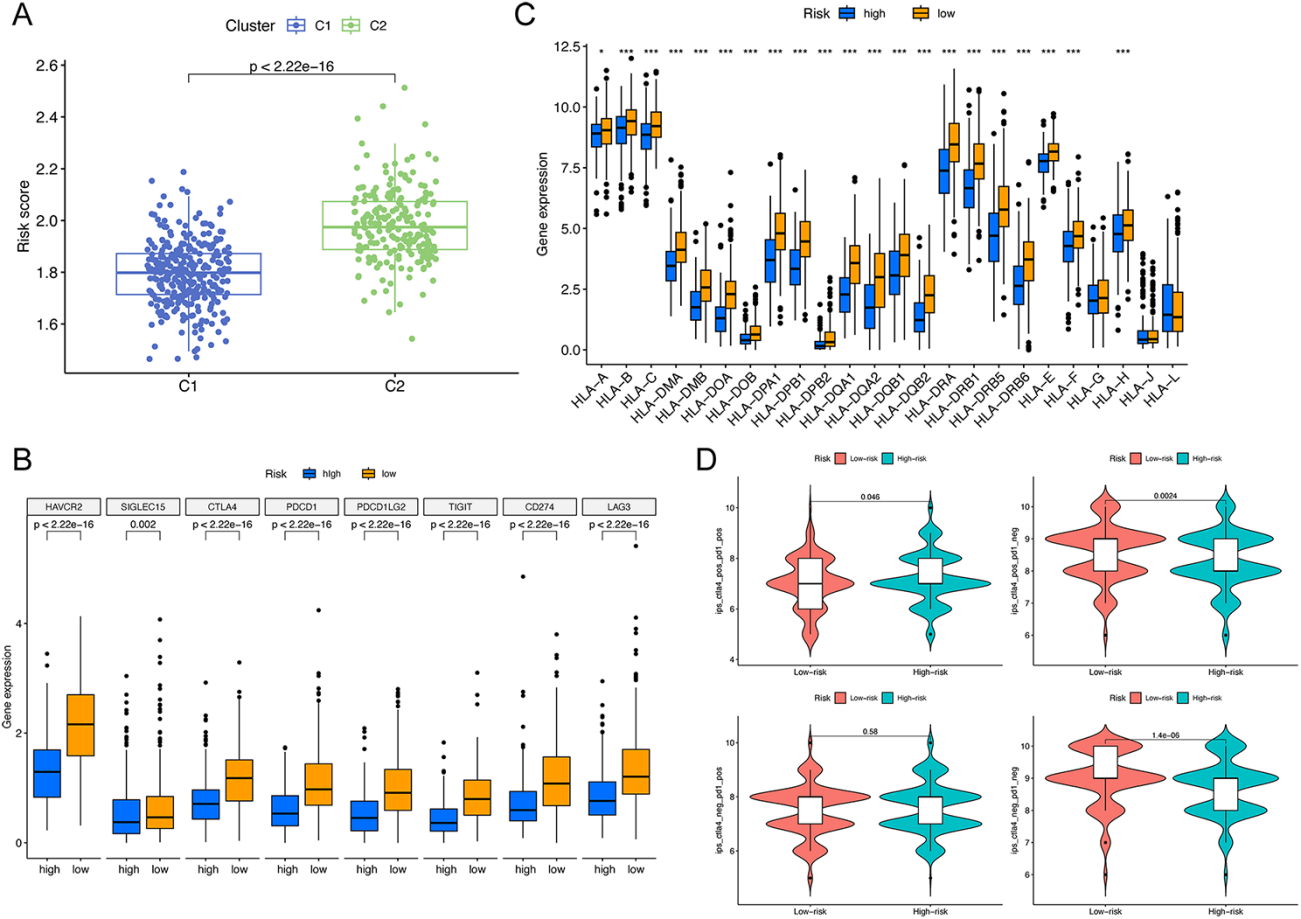
Prediction of response to immunotherapy. (**A**) In comparison to C1, C2 showed a higher risk score. (**B-C**) Difference in ICI- and HLA-related genes between clusters. (**D**) IPS score predicting immunotherapy response. ICI, immune checkpoint inhibitor; HLA, human leukocyte antigen; IPS, immunophenoscore. *P < 0.05; **P < 0.01; ***P < 0.001

Further investigation revealed that C2 presented with a higher risk score compared to C1 (Fig. 10A). In the search of identifying prospective treatment targets, we found that the HRG was characterized by a reduced expression of genes related to both ICI and HLA (Fig. 10B, C). By contrast, the LRG patients demonstrated a heightened IPS, suggesting potentially greater sensitivity to immunotherapies (Fig. 10D).

### qPCR

**Fig. 11:**
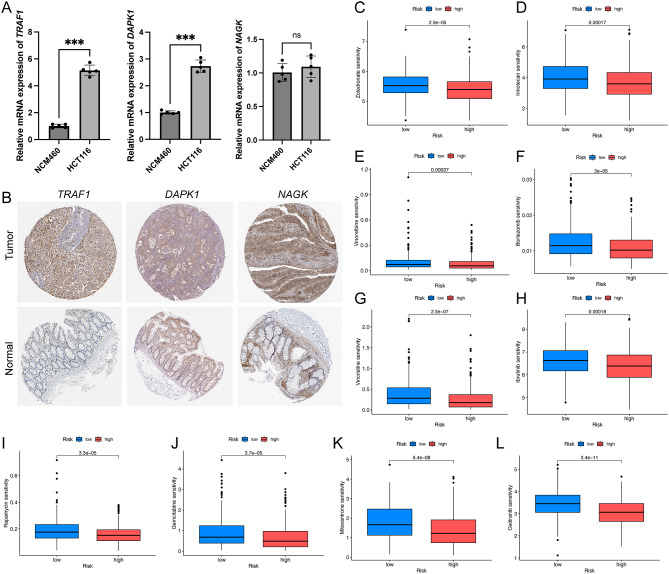
Experimental validation and anti-cancer drugs prediction. (**A**) qPCR. (**B**) IHC analysis from HPA database. (**C-L**) Anti-cancer drugs prediction. qPCR, Quantitative Polymerase Chain Reaction; IHC, immunohistochemistry; HPA, human protein atlas. *P < 0.05; **P < 0.01; ***P < 0.001

Using qPCR, we were able to assess the relative expression levels of mRNA in CRC cells. The findings of this study revealed a notable upregulation of TRAF1 and DAPK1 expression in CRC cells (Fig. 11A).

### IHC

For further validation, we consulted the HPA database, which corroborated the transcriptional patterns and supplemented them with protein expression data derived from IHC staining (Fig. 11B). Employing the oncoPredict algorithm, our analysis identified ten anticancer drugs (bortezomib, cediranib, gemcitabine, ibrutinib, irinotecan, mitoxantrone, rapamycin, vincristine, vinorelbine, and zoledronate) with reduced IC50 levels in the HRG (Fig. 11C-L). This suggests a heightened likelihood of therapeutic efficacy for these drugs in patients classified within the HRG.

## Discussion

CRC exhibits significant heterogeneity in clinicopathological and molecular profiles, influencing tumor progression and treatment responses [21]. TME interactions facilitate CRC progression via multiple pathways. TAMs play a pivotal role in tumor progression by mediating immunosuppression, extracellular matrix remodeling, and releasing growth factors [22]. TAMs also interact with various immune cells within the TME, aiding in immune suppression and facilitating tumor immune evasion [11]. The critical role of TAMs in CRC progression has sparked interest in TAM-targeted therapeutic strategies [23, 24]. Advances in immunotherapy have led to significant progress in cancer treatment [25]. However, not all patients respond to immunotherapy, largely due to TME characteristics [26]. PD-1 inhibitors have been shown to target TAMs directly, enhancing their phagocytic abilities [27]. Specific TAM markers, like CD40, show promise for use in adoptive cell immunotherapy [28]. Additionally, studies have demonstrated that CSF1R inhibitors can decrease TAM levels in the TME and induce macrophage repolarization towards the M1 phenotype, offering considerable clinical potential [29]. Eliminating SPP1 + TAMs could improve the efficacy of myeloid-targeted immunotherapy or enhance outcomes when combined with ICI therapies [9]. Despite these advancements, research in CRC remains insufficient, and further detailed studies on TAMs’ role in CRC prognosis are urgently needed.

In this study, we utilized bulk RNA-seq data to explore the prognostic implications of M2 macrophage infiltration in TCGA-CRC samples. Our findings indicate that higher levels of M2 macrophage infiltration are linked to poorer outcomes in CRC, underscoring the pivotal role of M2 macrophages in the prognosis. Acknowledging the dual roles of M2 macrophages in immunosuppression and tumor promotion, our study involved an intersectional analysis of M2 macrophage-associated genes from the TCGA-CRC dataset with TAM marker genes derived from scRNA-seq datasets [5, 6]. This integrative approach led to the discovery of 377 genes that are related to both M2 macrophages and TAMs. Subsequent univariate and LASSO Cox regression analyses facilitated the selection of three prognostic genes (DAPK1, NAGK, and TRAF1) for the construction of the TAMM2RS. DAPK1 is noteworthy for its specificity in anal squamous cell carcinoma and potential as a molecular biomarker [30]. Methylation of DAPK1 correlates with nodal metastasis and is considered a significant risk factor in CRC plasma [31, 32]. The DAPK1-ERK1 signaling axis is implicated in CRC metastatic progression, positioning DAPK1 as a key anti-metastatic factor and a prospective predictive biomarker [33]. Furthermore, inhibiting DAPK1 enhances cancer stem cell (CSC) stemness and the epithelial-mesenchymal transition (EMT) process, with the DAPK1-ZEB1 axis potentially intersecting the TGF-β and WNT pathways and influencing both CSCs and EMT processes [34]. TRAF1, on the other hand, is targeted by miR-483, a suppressor in colorectal cancer that hampers cell proliferation and migration [35]. Moreover, TRAF1 plays a role in the mobility and M1 polarization of macrophages, a process mediated by TNFSF9/TRAF1/p-AKT/IL-1β signaling in response to F. nucleatum AI-2 [36]. This comprehensive analysis elucidates the multifaceted roles of these genes in CRC, providing valuable insights for future research and potential therapeutic strategies.

In the field of CRC research, there has been a growing interest in developing various risk assessment models. Zhang et al. focused on crucial lysosome-related genes integral to CRC, thereby establishing a corresponding risk signature [37]. Similarly, Han and colleagues delved into adipogenesis-associated genes, creating a prognostic model while illuminating the immunogenomic landscape of CRC [38]. In another vein, Huang et al. explored genes with prognostic significance from the angle of fatty acid metabolism, suggesting their potential relevance in immunotherapy strategies [39]. These studies collectively enhance the accuracy of CRC prognosis predictions through diverse methodologies, demonstrating their models’ effectiveness. Conventional bulk RNA-seq methods provide an overall gene expression profile at the tissue level, yet fail to discriminate the transcriptomic diversity of various cell types and their proportions within these tissues. In a novel approach, our study integrates scRNA-seq data, which offers precise cell type identification and high-resolution expression profiles, with bulk RNA-seq data. This integration allowed us to pinpoint specific TAM-M2 prognostic biomarkers for CRC. To our knowledge, this is the first study to integrate these two data types for the purpose of identifying TAM-M2-related genes and developing a risk signature in CRC. The identification of these signature genes opens new avenues for deeper comprehension and exploration in CRC research. Moreover, our discovered prognostic signature holds promise for enhancing the clinical management of CRC patients.

Despite the encouraging outcomes of our research, it is important to acknowledge some inherent limitations. Firstly, the reliance on data sourced from public databases could potentially limit the representativeness of our findings across the broader patient demographic. Secondly, our conclusions are predominantly based on bioinformatics analyses, underscoring the need for further validation through detailed studies of molecular mechanisms. Consequently, there is a compelling necessity for more comprehensive investigations to elucidate the intricate roles of TAM-M2-related genes in CRC.

## Conclusion

In our study, we combined scRNA-seq and bulk RNA-seq analyses to unveil the diverse landscape of CRC at both the individual cell and tissue levels, culminating in the development of the TAMM2RS. This approach sheds new light on the multifaceted nature of M2 macrophages and TAMs, contributing to a deeper understanding of the TME complexities. Furthermore, our research identifies potential therapeutic targets for CRC, offering novel avenues for treatment strategies.

### Electronic supplementary material

Below is the link to the electronic supplementary material.

Supplementary Material 1

Supplementary Material 2

## Data Availability

The datasets employed in this research are available in the GEO (GSE132465, https://www.ncbi.nlm.nih.gov/geo/) and TCGA (TCGA-CRC, https://portal.gdc.cancer.gov/) repositories. For additional information, correspondences can be addressed to the authors responsible.
