# Supplementary material for: Integrated analysis of tumor-associated macrophages and M2 macrophages in CRC: unraveling molecular heterogeneity and developing a novel risk signature
Source: BMC Med Genomics. 2024 May 27;17:145. doi: 10.1186/s12920-024-01881-z (PMC11129467; doi:10.1186/s12920-024-01881-z)
Supplement: Supplementary file 2 — Supplementary Material 2 [file 12920_2024_1881_MOESM2_ESM.docx]

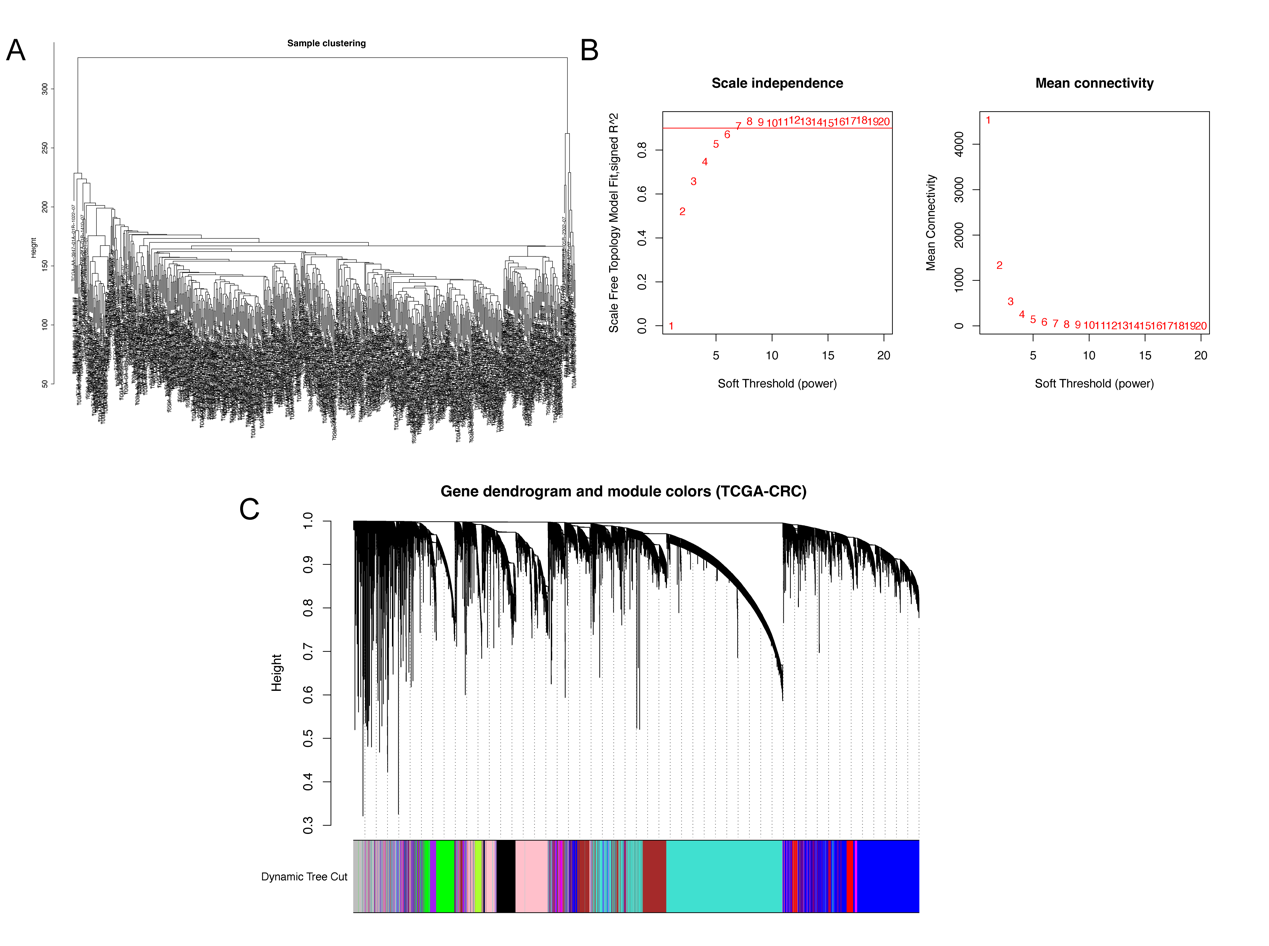
**Figure S1** Processing of WGCNA. (A) Sample clustering for outlier detection. (B) Selection of soft threshold power of 6 and assessment of average connectivity across a range from 1 to 20. (C) Construction of cluster dendrogram using genes with similar expression patterns. WGCNA, weighted gene co-expression network analysis.

**Figure S2** Cells from GSE132465 dataset were classified into 39 clusters.
